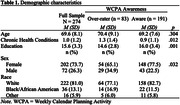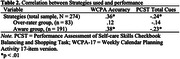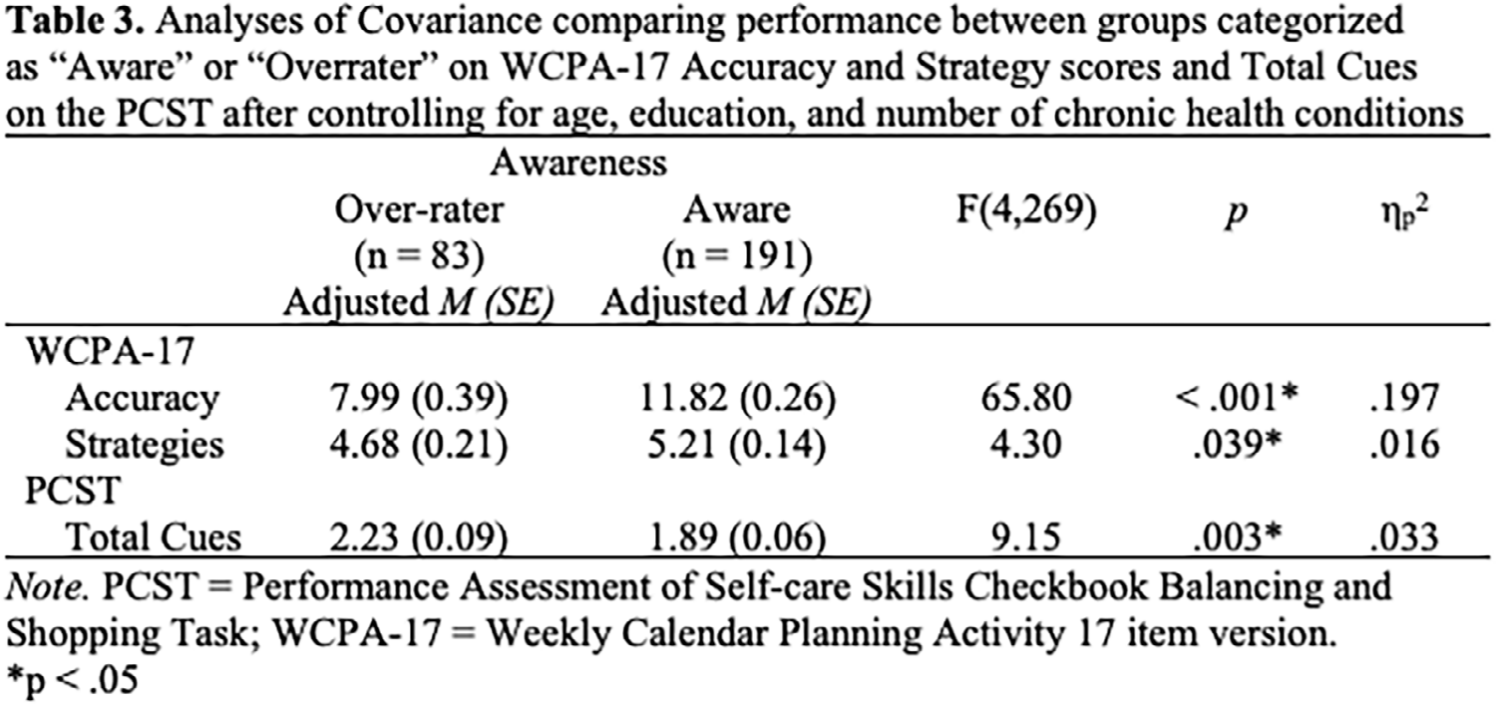# Online awareness: A key element of functional cognition

**DOI:** 10.1002/alz.089207

**Published:** 2025-01-09

**Authors:** Timothy S Marks, Dorothy F. Edwards

**Affiliations:** ^1^ University of Missouri ‐ Columbia, Columbia, MO USA; ^2^ Wisconsin Alzheimer’s Disease Research Center, University of Wisconsin School of Medicine and Public Health, Madison, WI USA; ^3^ University of Wisconsin Program in Occupational Therapy, Madison, WI USA

## Abstract

**Background:**

Intact awareness of impaired task performance can facilitate the adoption of cognitive strategies to support performance. However, most studies have not examined awareness of task performance (an element of metacognition) relative to complex everyday activities. The Weekly Calendar Planning Activity (WCPA) is a performance‐based assessment of functional cognition that includes indicators of awareness during performance of a simulated complex instrumental activity of daily living (IADL). This study examined if community‐dwelling adults’ Accuracy (primary outcome) and Strategy scores on the WCPA differ according to the individual’s awareness of task performance. Additionally, we examined whether functioning on the Performance Assessment of Self‐care Skills Checkbook Balancing and Shopping Task (PCST) as indicated by the Total Cues was associated with awareness on the WCPA.

**Method:**

Using data collected as part of a larger study we performed a cross‐sectional analysis of 274 community‐dwelling adults aged 55 to 93 years. Online awareness was evaluated on the 17‐item version of the WCPA (WCPA‐17) using participant subjective ratings of performance compared to actual observed performance. Participant data were classified into two groups: those who were aware of their performance versus those who over‐rated performance. A one‐way Analysis of Covariance was used to test three dependent variables, Accuracy scores on the WCPA‐17, number of Strategies used on the WCPA‐17, and Total Cues on the PCST, against the bimodal awareness state while controlling for age, education, and number of chronic health conditions as covariates.

**Result:**

Individuals categorized as aware of their performance on the WCPA‐17 had significantly better Accuracy scores on the WCPA‐17 (F(4,269) = 65.80, p < .001), used more Strategies on the WCPA‐17 (F(4,269) = 4.30, p = .039), and needed fewer Total Cues on the PCST (F(4,269) = 9.15, p = .003).

**Conclusion:**

Overall, the group categorized as aware on the WCPA‐17 performed better on all functional cognition performance scores. Intact online awareness is critical to performance on complex IADLs and can be evaluated with functional cognition assessments. The WCPA‐17 can be used to evaluate awareness in the context of complex IADL and may be useful for assessment and intervention planning.